# EnSVMB: Metagenomics Fragments Classification using Ensemble SVM and BLAST

**DOI:** 10.1038/s41598-017-09947-y

**Published:** 2017-08-25

**Authors:** Yuan Jiang, Jun Wang, Dawen Xia, Guoxian Yu

**Affiliations:** 1 College of Computer and Information Science, Southwest University, Chongqing, China; 2 College of Data Science and Information Engineering, Guizhou Minzu University, Guiyang, China; 3 College of National Culture and Cognitive Science, Guizhou Minzu University, Guiyang, China

## Abstract

Metagenomics brings in new discoveries and insights into the uncultured microbial world. One fundamental task in metagenomics analysis is to determine the taxonomy of raw sequence fragments. Modern sequencing technologies produce relatively short fragments and greatly increase the number of fragments, and thus make the taxonomic classification considerably more difficult than before. Therefore, fast and accurate techniques are called to classify large-scale fragments. We propose EnSVM (*En*semble *S*upport *V*ector *M*achine) and its advanced method called EnSVMB (*EnSVM* with *B*LAST) to accurately classify fragments. EnSVM divides fragments into a large confident (or small diffident) set, based on whether the fragments get consistent (or inconsistent) predictions from linear SVMs trained with different *k*-mers. Empirical study shows that sensitivity and specificity of EnSVM on confident set are higher than 90% and 97%, but on diffident set are lower than 60% and 75%. To further improve the performance on diffident set, EnSVMB takes advantage of best hits of BLAST to reclassify fragments in that set. Experimental results show EnSVM can efficiently and effectively divide fragments into confident and diffident sets, and EnSVMB achieves higher accuracy, sensitivity and more true positives than related state-of-the-art methods and holds comparable specificity with the best of them.

## Introduction

Metagenomics, directly collected from natural environments, can be used to characterize genome sequences in microbial communities. Metagenomics provides a new approach to explore the microbial bio-diversity and the microbial communities, which are not available in human-cultured environments^1^. On the other hand, metagenomics brings in new computational challenges, i.e., the need for assembly and gene-finding programs to handle highly diverse sequence collections of species and tools to accurately classify large scale sequence fragments.

Metagenomics fragments classification is to assign a fragment to a corresponding species (or taxonomy). Many computational methods have been proposed to automatically determine the taxonomy of fragments. These methods can be roughly divided into two categories: alignment-based and composition-based. Alignment-based methods use alignment tools (i.e., BLAST^2^) to align fragments to known reference sequences and assign fragments to a species based on the best match^3, 4^. For example, MEGAN^5^ classifies fragments based on multiple high-scoring BLAST hits, it assigns fragments to the lowest common ancestor of those BLAST matches that over a bit-score threshold. BWA^6^ is another popular alignment package that aligns fragments against a large amount of reference sequences, such as human genome and other microbial genomes. Composition-based methods usually assign fragments based on their *k*-mer signatures. Two basic ideas are widely adopted in compositional approaches. (i) Compositional methods utilize *k*-mer index schemes to construct a reference *k*-mer database and assign fragments by referring to the most similar *k*-mer sets. Kraken is a representative method of *k*-mer index scheme^7^. (ii) Compositional approaches based on machine learning models use *k*-mer profiles as input, such as interpolated Markov models (IMMs)^8, 9^, *k*-nearest neighbors (*k*NN) classifier^10^, naive Bayesian classifier (NBC)^11–13^, support vector machine (SVM)^14–16^ and so on. Phymm utilizes IMMs to characterize variable-length *k*-mers of a phylogenetic group and then handles the general phylogenetic classification problem^8^. PhymmBL^8, 9^, a hybrid method based on Phymm and BLAST, combines the results of BLAST with scores produced by IMMs to improve the accuracy of using BLAST alone. Traditional *k*NN is faced with the curse of dimensionality problem when the dimensionality of *k*-mer profiles is high^10^. To solve this problem, TACOA^10^ introduces a Gaussian kernel to extend the traditional *k*NN and applies *k*NN for fragments classification. PhyloPythia^14^ takes the oligonucleotide composition of variable-length genome fragments as input data for SVM. To adapt SVM for multi-class classification, it applies the ‘all-versus-all’ technique and trains *C*(*C* − 1))/2 (*C* is the number of species) binary SVMs, one for each pairwise combination of two species, then it assigns a fragment to a species based on the votes aggregated from these SVMs. Different from PhyloPythia that uses binary SVMs, PhyloPythiaS^15^, a successor of PhyloPythia, adopts a structural SVM to classify fragments.

DNA of different species are not the same, but there are some similar DNA fragments from different species, especially for short fragments sequenced by modern sequencing techniques^17, 18^. Short fragments increase the risk that fragments from different species have similar DNA segments, which are hard-to-be classified by most compositional methods. Therefore, the accuracy of these methods is significantly impacted.

In this study, we introduce an accurate and efficient metagenomics fragments classification method called EnSVM (*En*semble *SVM*) and its advanced method called EnSVMB (*EnSVM* with *B*LAST). Most composition-based methods^8, 10, 13–16, 19^ only use one fixed *k*-mer for classification, which results in information loss with such single *k*. Furthermore, choosing an applicable and effective *k* for these *k*-mer based methods is troublesome. Different from these methods^8, 10, 13–16, 19^, EnSVM trains several linear SVMs with different *k*-mers and combines these SVMs into an ensemble classifier. To differentiate short fragments that are *easy-to-be* (or *hard-to-be*) classified, EnSVM divides a fragment into the *confident* (or *diffident*) set based on whether the fragment obtains consistent (or inconsistent) predictions from these SVMs. Empirical study shows that the sensitivity and specificity of EnSVM on fragments in the confident set are higher than 90% and 97%, and those on the diffident set are lower than 60% and 75%. To improve the performance and retrieve more true positive fragments from the diffident set, EnSVMB, an advanced method of EnSVM, resorts to BLAST (blastn)^20^ to further classify fragments in the diffident set. For a query fragment, if BLAST cannot retrieve a relevant sequence with best hit and confident *e*-value from the reference set, EnSVMB tags this fragment as unknown. Empirical study on a small metagenomics dataset shows that EnSVMB improves the accuracy of EnSVM on the diffident set from 54.95% to 88.85%, sensitivity from 60.37% to 87.55% and specificity from 50.23% to 98.12%. Further study on a medium one, a large one and a simulated dataset with noise demonstrates that EnSVMB obtains higher accuracy, sensitivity and more true positives than related state-of-the-art methods, and EnSVMB holds comparable specificity with BWA and BLAST. Results on a real gut metagenome also corroborate these advantages of EnSVMB.

## Results

### Preliminary investigation on a small metagenomics dataset

In this section, we investigate the rationality of EnSVM and EnSVMB and study the effect of different *k*-mers. For this purpose, we use a small dataset (details are described in Section Material), which includes about 257,000 short fragments in the reference set, and 82,876 fragments in the validation set.

At the beginning, we make use of seven linear SVMs to study the effect of different *k*-mers with *k* fixed as one value among {5, 6, 7, 8, 9, 10, 11}. We use the default parameters of LIBLINEAR^21^ to train these SVMs on the reference set and then test these SVMs on the validation set. As Table 1 shown, the accuracy, sensitivity, specificity and the number of true positives are rising as the increase of *k*, but when *k* is larger than 8, they gradually decrease. This fact suggests a large *k*-mer does not necessarily result in better performance, although a large *k*-mer significantly increases the amount of *k*-mer profiles. We can find that accuracy, sensitivity and specificity of linear SVMs with $k\in \{6,7,8,9,10\}$ are higher than those of $k\in \mathrm{\{5},\mathrm{11\}}$ and closed to each other. Therefore, EnSVM chooses to aggregate the predictions of linear SVMs with $k\in \{6,7,8,9,10\}$. In practice, the accuracy (90.67%), sensitivity (90.81%), specificity (89.79%) and the number of true positives (75144) of EnSVM on the validation set are larger than that of any linear SVM.

**Table 1:** Results of linear SVM on validation set with different *k*-mers.

Methods	Accuracy	Sensitivity	Specificity	True positives
SVM (*k* = 5)	86.95%	85.62%	86.77%	72086
SVM (*k* = 6)	86.34%	88.17%	89.21%	74041
SVM (*k* = 7)	90.39%	90.37%	89.37%	74912
SVM (*k* = 8)	90.46%	90.58%	89.57%	74970
SVM (*k* = 9)	88.96%	89.21%	88.43%	73726
SVM (*k* = 10)	89.49%	89.85%	89.17%	74166
SVM (*k* = 11)	83.95%	84.23%	85.54%	69574

Accuracy is computed as the ratio between the number of true positives and the number of fragments in the validation set^8^.

EnSVM firstly divides fragments in the validation set into the confident or diffident sets by voting mechanism. Particularly, fragments in the confident set ge consistent votes ($vote=5$), whereas fragments in the diffident set get inconsistent votes from these linear SVMs. As Table 2 shown, the accuracy, sensitivity and specificity of EnSVM on confident set (71496 fragments) are 95.12%, 95.47% and 97.10%, whereas those on diffident set (11380 fragments) are only 54.95%, 60.37% and 50.23%. The large accuracy, sensitivity and specificity margin on the confident set and diffident set indicates that EnSVM can pick out the hard-to-be classified fragments by aggregating predictions from five linear SVMs. By dividing the fragments into confident and diffident sets, we can separately treat these two sets and pay more attention to the diffident set. Here, we use an advanced method of EnSVM called EnSVMB to further classify fragments in the diffident set. Particularly, EnSVMB uses BLAST (with default *e*-value) to reclassify fragments in the diffident set. For a fragment in the diffident set, if BLAST cannot find a sequence with best BLAST hit and confident *e*-value from the reference set, EnSVMB tags this fragment as unknown. Table 2 shows the results and runtime of EnSVM and EnSVMB in different stages. EnSVMB improves the accuracy, specificity and number of true positives of EnSVM on diffident set. The accuracy on the diffident set is 88.85%, sensitivity is 87.55%, specificity is 98.12% and number of true positives of EnSVMB is 8272, whereas those of EnSVM are 54.95%, 60.37%, 50.23% and 6253. EnSVMB only resorts to BLAST on diffident set, which accounts for a small portion of fragments in the validation set, so it does not bring in significantly increased runtime by additionally running BLAST. In fact, EnSVMB on the validation set only takes 11 min 54 s (5 min 21 s for EnSVM on the validation set and 6 min 33 s for BLAST on the diffident set). In contrast, BLAST (parallel on 6 CPU cores) on the validation set asks for 45 min 3 s, and its accuracy, sensitivity, specificity and number of true positives are 85.21%, 89.01%, 98.15% and 70624 respectively.

**Table 2:** Accuracy, sensitivity, specificity, number of true positives and runtime of EnSVM and EnSVMB on different stages. Experiment platform configuration: CentOS 6.5, Intel Xeon E5-2678v3 and 256GB RAM.

Stage	Accuracy	Sensitivity	Specificity	True positives	Runtime
EnSVM (confident set 71496 fragments)	95.12%	95.47%	97.10%	68886	5 min 21 s
EnSVM (diffident set 11380 fragments)	54.95%	60.37%	50.23%	6253	5 min 21 s
EnSVMB (diffident set 11380 fragments)	88.85%	87.55%	98.12%	8272	6 min 33 s
EnSVMB (validation set 82876 fragments)	95.32%	94.06%	97.76%	77158	11 min 54 s

1st row are the results of EnSVM on the confident set (71496 fragments). 2nd row are the results of EnSVM on the diffident set (11380 fragments). 3rd row are results of EnSVMB on the diffident set and BLAST parallel runs on 6 CPU cores. 4th row is the prediction results of EnSVMB on the validation set.

### Results on a medium dataset

In this section, we explore the performance of EnSVM and EnSVMB on a medium dataset. Furthermore, we also evaluate the performance of EnSVMB with different parameters. Medium dataset includes two sets: reference set and validation set (details are described in Section Material). Both reference set and validation set are represented by five *k*-mer profiles ($k\in \{6,7,8,9,10\}$), and there are about 10^6^ fragments in the reference set and about 2.7 × 10^5^ fragments in the validation set.

To comparatively and quantitatively study the performance of EnSVM and EnSVMB, we consider three compositional methods Kraken^7^, VW (Vowpal Wabbit)^19^ and NBC (Naive Bayes Classifier)^13^, two alignment-based methods BWA (Burrows-Wheeler Alignment tool)^6^, BLAST (blastn)^20^ as comparing methods. We also record the actual runtime cost of these methods. In fact, we had tried non-linear SVM, but non-linear SVM did not complete in 7 days, so the results of non-linear SVM are not reported here. For linear SVMs, the constraints violation loss parameter $\omega$ is set as 32 by 5-fold cross-validation on the reference set. Parameter *k* for VW and NBC are set as 10 and other parameters are fixed as the default values as the author suggested or provided in the codes. Voting threshold of EnSVM is set to 5.

Table 3 reports the results of different methods with respect to accuracy, sensitivity, specificity and the number of true positives. We first note that VW and NBC are outperformed by EnSVM and EnSVMB on almost all metrics. The possible reason is that NBC and VW are two compositional methods and only use a fixed *k*-mer size. Some fragments from different species may have the similar (or the same) *k*-mer profiles under a particular *k*-mer size. On the other hand, these fragments may have different *k*-mer profiles under other *k*-mers. EnSVM and EnSVMB consider five different *k*-mers and employ more profiles than VW and NBC. Five linear SVMs trained with different *k*-mers form a committee that helps to more accurately classify fragments than using any of them alone. In addition, EnSVMB further deals with the fragments in the diffident set to increase the number of true positives fragments. It is worth noting that the specificity of BWA and BLAST is slightly higher than EnSVMB. The cause is that BWA and BLAST utilize sequence alignment to exhaustively search all fragments in the reference set. This exhaustive search enables them to correctly classify fragments that have similar fragments in the reference set. Nevertheless, the accuracy, sensitivity and number of true positives of BWA and BLAST are much lower than those of EnSVMB. That is principally because alignment-based methods sometimes cannot find out similar sequences from the reference set, and the query fragment may be mutated or with sequence error. For these reasons, the accuracy, sensitivity and number of true positives of alignment-based methods drags down. Kraken, a widely used *k*-mer index method, is also outperformed by EnSVM and EnSVMB. The reason is that Kraken heavily depends on the adopted *k*-mer sets to represent a lineage, which are chosen by professionals. However, choosing accurate and representative *k*-mer sets is a non-trivial job and asks for a lot of time. These observations again corroborate the advantage of EnSVM and EnSVMB for fragments taxonomy classification. We also list the results of EnSVM on the diffident set, and its accuracy (64.09%), sensitivity (67.13%) and specificity (75.29%) are much lower than those on confident set (97.83%, 94.14% and 97.12%). The performance margin on confident and diffident sets shows that EnSVM can accurately classify fragments in the confident set and pick out hard-to-be classified fragments. Apart from specificity, EnSVM gets higher accuracy, sensitivity and more true positives than other comparing methods (except EnSVMB) on the validation set. This observation indicates that ensemble classifier can be a competitive alternative tool for taxonomy classification of fragments.

**Table 3:** Results on a medium metagenomics dataset in species level.

Methods	Accuracy	Sensitivity	Specificity	True positives	Training time	Prediction time
VW	85.24%	84.63%	90.11%	201146	401 min 09 s	22 min 45 s
NBC	75.45%	76.52%	83.54%	203405	1 min 52 s	20 s
Kraken	84.33%	80.03%	95.60%	227344	—	1 min32 s
BLAST(blastn)	83.71%	82.81%	**98.17**%	225673	—	1773 min 36 s
BWA	81.57%	78.80%	**99.75**%	213204	—	38 min 14 s
EnSVM on confident						
set (175985)	97.83%	94.14%	97.12%	175985	51 min 3 s	10 min 20 s
EnSVM on diffident						
set (93604)	64.09%	67.31%	75.29%	59991	51 min 3 s	10 min 20 s
EnSVM	86.12%	84.44%	90.52%	232170	51 min 3 s	10 min 20 s
EnSVMB	**88.04**%	**86.48**%	**98.12**%	**237346**	51 min 3 s	35 min 14 s

Although VW only uses a single *k*-mer, it still takes longer training time than EnSVM and EnSVMB. The reason is that VW needs to iteratively optimize the model. NBC assumes the attributes of *k*-mer profiles are independent from each other, and Kraken uses pre-indexed *k*-mer sets. Therefore, they run faster than other comparing methods. The total runtime of BWA is a little smaller than EnSVM and EnSVMB. The reason is that BWA applies a fast backward search with Burrows Wheeler Transform^22^ and supports multiple sequences alignment, but its runtime in the prediction stage is longer than EnSVM and EnSVMB. Since BLAST query each fragment in the validation set, it runs much slower than all the other comparing methods.

We also evaluate these methods in phylum level (results are reported in Table 4). We can find that EnSVMB has the highest accuracy and sensitivity, largest number of true positives. BLAST, BWA and EnSVMB have comparable specificity and their specificities are higher than other methods. It is obvious that almost all methods in phylum level outperform themselves in species level. That is because classification in phylum level is easier than in the species level. The results in Table 4 and other tables demonstrate that EnSVM and EnSVMB can not only work well in species level, but also can in other levels.

**Table 4:** Results on a medium metagenomics dataset in phylum level.

Methods	Accuracy	Sensitivity	Specificity	True positives
VW	85.12%	86.11%	92.42%	229474
NBC	79.32%	76.64%	84.65%	213838
BLAST (blastn)	85.44%	86.65%	**99.96%**	230337
BWA	84.23%	82.36%	**99.23%**	227074
Kraken	86.71%	89.36%	98.63%	233760
EnSVM	87.78%	85.54%	90.04%	236645
EnSVMB	**92.10%**	**90.42%**	**99.02%**	**248291**

**Figure 1:**
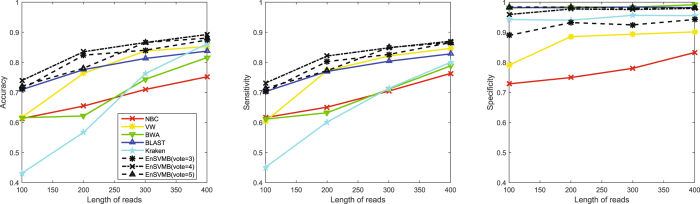
The performance of six methods under different lengths of fragments. Particularly, EnSVMB(vote = 3), EnSVMB(vote = 4) and EnSVMB(vote = 5) means that the voting threshold of EnSVMB is set as 3, 4 and 5, respectively.

The typical length of short reads is between 75 to 400 bp. To study the performance of EnSVMB in this range, we increase the length of short reads from 100 to 400 with stepsize of 100 and report the results in Fig. 1. In addition, we also investigate the performance of EnSVMB under different voting thresholds (3, 4 and 5).

From these results, we can see that the performance of all methods increases as the length increasing. The possible reason is that compared with long fragments, short fragments usually contain less information and are more difficult to be classified. We can also find that EnSVMB performs better than VW, NBC, BWA, Kraken and BLAST on accuracy and sensitivity under different voting thresholds. However, an interesting observation is that EnSVMB with voting threshold 4 is better than the counterpart with voting threshold 5 when the length of fragments in the range 100–200 bp. This is principally because the confident set under voting threshold 4 includes about 70% fragments, whereas the corresponding confident set under voting threshold 5 includes no more than 50% fragments. If we set the voting threshold too strict, a large portion of fragments with high confident are assigned to the diffident set and they maybe misclassified by alignment based tools. But this observation does not mean the smaller the voting threshold, the better the performance of EnSVMB is. If we set the voting threshold lower than 4, more fragments are assigned to the confident set but a number of fragments are wrongly assigned to this set, so the specificity of EnSVMB is significantly declined. From these observations, we suggest that when the length of short reads is small, a moderate threshold should be adopted; in other cases, a large threshold is better.

Kraken, NBC and VW are three *k*-mer composition-based methods, all of them are significantly outperformed by BWA, BLAST and EnSVMB. Kraken heavily depends on the adopted *k*-mer sets, so it cannot work well if there are not enough *k*-mer sets. NBC and VW only use a fixed *k*-mer size and utilize less information than EnSVMB. These results show that EnSVMB can achieve higher accuracy, sensitivity than related state-of-the-art methods in the typical length of short reads, and EnSVMB can hold comparable specificity with the best of them.

### Results on a large dataset

In this section, we further evaluate the performance of VW, NBC, BLAST, BWA, Kraken and EnSVMB on a large dataset, which contains 1702 complete genome sequences from 331 species (details are described in Section Material). The corresponding validation set includes 184 complete genome sequences not present in the reference set but originated from these 331 species.

**Figure 2:**
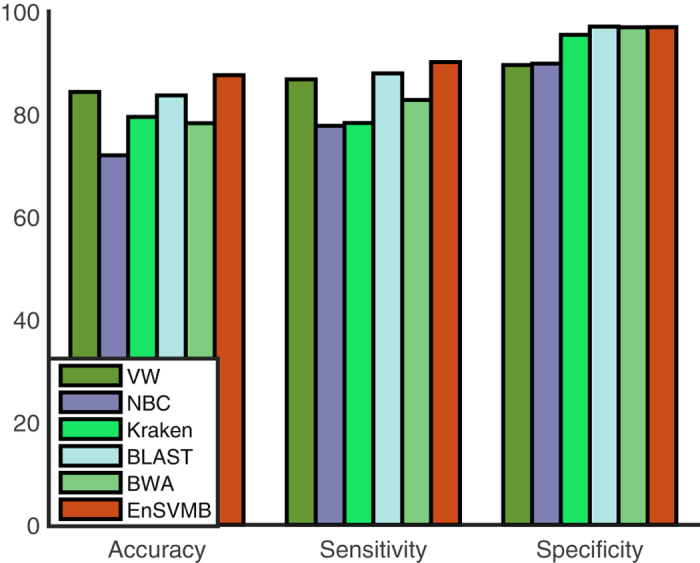
The performance of six methods on large-scale dataset.

Figure 2 shows the results of six methods on this large dataset with respect to accuracy, sensitivity and specificity. By referring to the results on the medium dataset, the accuracy of VW decreases from 85.24% to 84.29%, NBC from 75.45% to 71.95%, Kraken from 84.33% to 79.45%, BWA from 81.57% to 78.20%, BLAST from 83.71% to 83.60%, and EnSVMB from 88.04% to 87.36%. This decrease pattern is because it is more difficult to accurately classify as the number of species increasing. As Fig. 2 shown, EnSVMB obtains highest accuracy, with about 9% and 3% higher than BWA and BLAST, respectively. As well as that, EnSVMB has the highest sensitivity and holds comparable specificity with BWA and BLAST (96.92% vs. 96.89% vs. 96.96%). Results on large dataset are consistent with the results of these methods on the medium dataset. From these results, we can conclude that EnSVMB can achieve competitive performance on large-scale dataset.

### Results on a simulated dataset

In the previous Subsections, we evaluate EnSVM and EnSVMB on DNA fragments obtained from NCBI reference genomes without errors. In real cases, sequencing errors may alter reads and make the classification problem more difficult. To evaluate the robustness of EnSVM and EnSVMB, we generate a simulated dataset with sequencing errors and mutations.

We use Grinder read simulation software^23^ to generate simulated validation set with median error rate of 2%. The simulated validation set is generated based on the validation set used in Subsection Results on a medium dataset, and the reference set keeps the same as the reference set used in Subsection Results on a medium dataset. Table 5 reveals the results of these comparing methods on the simulated dataset. Comparing with the results in Table 3, we can see the performance of BWA decreases about 0.5% on the simulated dataset under all metrics, NBC and EnSVM downgrade less than 0.3%. BLAST, EnSVMB and Kraken are the most robust methods, since they decrease less than 0.1%. These results suggest EnSVMB is robust to query fragments with mutations or errors.

**Table 5:** Results on a simulated metagenomics dataset.

Methods	Accuracy	Sensitivity	Specificity	True positives
VW	84.23%	83.79%	89.16%	198763
NBC	75.17%	76.29%	83.25%	202650
BLAST (blastn)	83.65%	82.73%	**98.27%**	225511
BWA	81.03%	78.34%	**99.23%**	218448
Kraken	84.25%	79.98%	95.52%	227129
EnSVM	85.82%	84.06%	90.21%	231361
EnSVMB	**88.01%**	**89.61%**	**98.01%**	**237265**

### Results on a real gut metagenome

**Figure 3:**
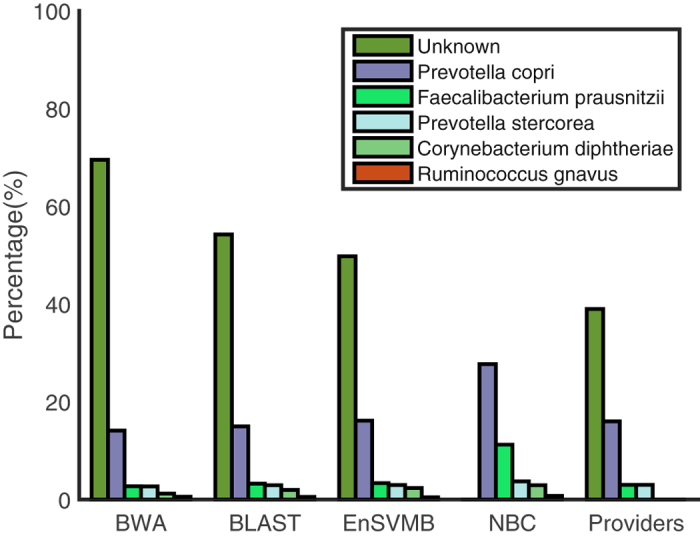
Abundance profiles identified by BWA, BLAST, EnSVMB and NBC. ‘Providers’ means that the abundance profiles are taken from EBI (https://www.ebi.ac.uk/metagenomics/).

We finally evaluate EnSVMB on a real gut metagenome downloaded from EBI metagenomics (https://www.ebi.ac.uk/metagenomics/). Project id of this metagenome is ERP014712, sample id is ERS1102103, run id is ERR1347146 and sequence data name is ‘processed nucleotide reads set’. We delete fragments whose length is less than 75 bp, since these fragments are too short to be classified. This metagenome has no certainty about knowledge of each individual read. For this reason, we list abundance profiles (with high abundance percentages in species level) provided by providers of this metagenome in Fig. 3. We also list the abundance profiles obtained by BWA, BLAST, EnSVMB and NBC in Fig. 3. Here, the reference set collected from NCBI RefSeq^24^ includes 80 species (details are listed in Supplementary Table S3). The voting threshold of EnSVMB is set as 4, the *e*-value of BLAST is set as 10^−5^ and identify percentage of BLAST is fixed as 97.5%.

From Fig. 3, we can see most fragments in this metagenome are tagged as unknown. EnSVMB tags 49.12% fragments as unknown, and BLAST and BWA tag much more fragments as unknown than EnSVMB. The reason is BLAST and BWA are two alignment based tools, they identify fragments by searching all sequences in the reference set. If the query fragment is mutated or with sequence errors, these tools may not be able to accurately identify related fragments. We also note that NBC does not tag any fragment as unknown and it obtains more abundance profiles than other methods. That is because that NBC mandatorily assigns each fragment to its most similar species. Prevotella copri in EnSVMB has very high abundance (16.11%), and it is highly comparable with the classification by the providers (16%). In contrast, Prevotella copri in BWA (14.11%) and BLAST (14.98%) are fewer than EnSVMB and the classification by the providers. Both Faecalibacterium prausnitzii (2.74% vs. 3.26% vs. 3.37% vs. 3%) and Prevotella stercorea (2.69% vs. 2.95% vs. 2.97% vs. 3%) in BWA, BLAST, EnSVMB and providers are highly comparable. It is worth noting that Ruminococcus gnavus and Corynebacterium diphtheriae are only displayed in BWA, BLAST, EnSVMB and NBC. The reason is that providers may not identify fragments in these species, abundance profiles of these fragments are not provided in species level and tagged as unknown. From these results, we can conclude that EnSVMB can accurately identify fragments in species level.

## Discussion

In this paper, we propose a new paradigm to accurately and efficiently classify metagenomics fragments. Our preliminary study shows that some fragments can be easily classified, but others are rather difficult to be correctly classified. That is principally because modern sequencing technologies produce a huge number of short fragments (or reads). Although these short fragments are from different species, they are too short to provide discriminative patterns^25^. Current efforts toward accurate short fragments classification resorts to various techniques^8, 10, 13–16, 19^, but most of them equally treat each fragment, and they do not discriminative the easy-to-be classified fragments from hard-to-be classified ones.

Inspired by these observations, we suggest two approaches called EnSVM and EnSVMB to accurately and efficiently classify short fragments. EnSVM and EnSVMB are motivated by the issue of choosing effective parameters for *k*-mer based classifiers in taxonomic classification of fragments and the advantage of ensemble learning, which often produces better performance on complex data than using a single classifier alone. EnSVM first trains five linear SVMs based on five different *k*-mer profiles, and then it accurately divides the easy-to-be (or hard-to-be) classified fragments into confident (or diffident) sets based on the aggregated predictions from these SVMs. Our study shows that the accuracy, specificity and sensitivity of EnSVM on the confident set are much higher than that on the diffident set. In fact, EnSVM can provide the option to construct two or even more sets with different voting thresholds, and enable further analysis of fragments in any of these sets to explore interesting patterns from them. The number of fragments in the confident set is much larger than that of diffident set. Thus, other alternative tools can be used to only reclassify these hard-to-be classified fragments, instead of all the fragments, and thus save much time.

EnSVMB integrates EnSVM with BLAST to put more emphasize on fragments in the diffident set and achieves significantly improved performance on these fragments. EnSVMB applies BLAST only on the diffident set. EnSVMB obtains higher accuracy, sensitivity and more true positive predictions than other comparing methods, and it has comparable specificity with BLAST and BWA. In addition, EnSVMB runs much faster than BLAST and VW, and it slightly slower than BWA.

In this investigative study, we only study the combination of *k*-mers in a fixed range, other combinations of *k*-mers may bring in even more accurate prediction and are worth for future investigation. Exploring other ways to divide fragments into different sets and then apply different techniques on different sets are interesting future pursues.

## Material

We evaluated EnSVM and EnSVMB in species level on three datasets. Each dataset comprises a set of reference genomes for training and a set of validation genomes for testing. We downloaded more than 5000 complete bacterial and archeal genomes from NCBI RefSeq^24^ database in September 2016. Next, we retain complete genomes of a species represented by at least three genomes, and then remove short sequences (genomes less than 10^6^ nucleotides). This preprocess follows the suggestions by Parks *et al*.^13^ and Vervier *et al*.^19^. After the filter process, 770 species are remained. Then, we generate three datasets (a small one, a medium one and a large one) from these species.

The small dataset is adopted for preliminary investigation of EnSVM and EnSVMB. The reference set used for training includes 47 complete genome sequences from 8 species. These 8 species are randomly chosen from 770 species. We also choose 18 complete genome sequences not present in the training database but originated from these 8 species as the validation set. The detail of this small set is listed in Table 6.

**Table 6:** Details of the small dataset.

Species	Number of genome sequences in the reference set
Corynebacterium diphtheriae	12
Brucella abortus	6
Methylobacterium extorquens	7
Lactobacillus rhamnosus	5
Erwinia amylovora	3
Shigella boydii	6
Desulfovibrio vulgaris	5
Bacteroides fragilis	3

As to the medium dataset, we randomly choose 69 species from 770 species as the reference set. 192 complete genome sequences from 69 species (details are listed in Supplementary Table S1) are collected as the reference set, and 64 complete genome sequences not present in the reference set but originated from these 69 species are used to validate the performance of EnSVMB and that of other comparing methods.

As to the large dataset, we randomly choose 331 species from 770 species as the reference set (details are listed in Supplementary Table S2), which includes 1702 complete genome sequences. The corresponding validation set includes 184 complete genome sequences not present in the reference set but originated from these 331 species.

Next-generation sequencing techniques improve the speed and reduce the cost on sequencing a genome. These techniques parallelize the sequencing process, produce thousands or millions of short fragments concurrently^17, 18^. Comparing with the old sequencing techniques, modern techniques produce relatively short fragments (75–400 base pairs)^8^. To adapt to modern techniques, each genome sequence is divided into short fragments of length 400 for experiments.

## Method

### EnSVM

Most composition-based classification methods use *k*-mer profiles to represent a fragment. The term *k*-mer typically refers to all the possible words of length *k* that are contained in a fragment. *k*-mer profiles are composed with numeric vectors, each entry of which counts the number of occurrences of a specific combination with A, T, C and G with length *k* in a sequence. Because there are 4 types of nucleotides in a fragment, each *k*-mer profile is a numeric vector with length 4^*k*^. Obviously, the length of this numeric vector increases exponentially as the increase of *k*. Note, for a fragment with length *l*, there are at most *l* − *k* + 1 non-zero entries in its *k*-mer profile vector. Almost all *k*-mer based methods^8, 10, 13–16, 19^ only utilize an applicable *k*-mer to balance the accuracy and efficiency in classifying fragments. They usually have to do a series of exploratory experiments to search an optimal *k*-mer, and they ignore complementary information encoded by sub-optimal *k*-mers.

**Figure 4:**
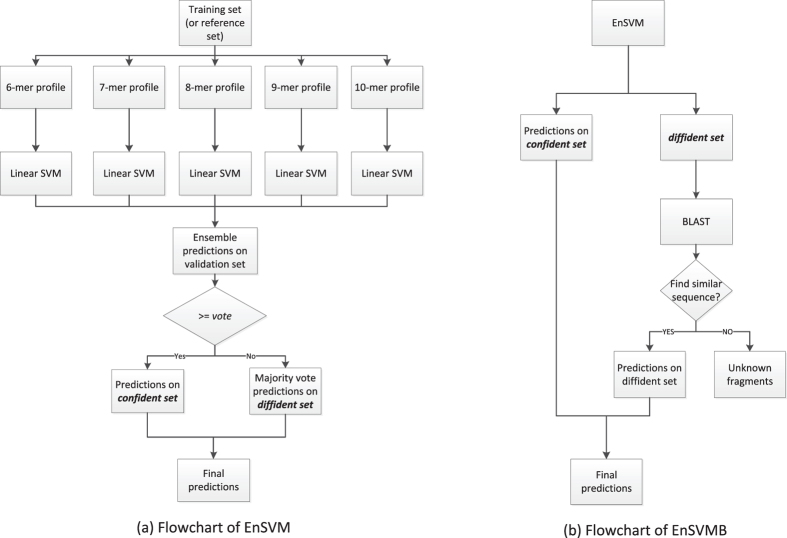
Five linear SVMs are integrated into an ensemble classifier (EnSVM). EnSVM then divides fragments in the validation set into the confident and diffident sets based on the aggregated predictions from these SVMs. The voting threshold (labeled as vote) is adjustable. EnSVMB further applies BLAST to reclassify fragments in the diffident set and tags fragments can not be retrieved from the reference set with confident *e*-value as unknown.

Different from these methods^8, 10, 13–16, 19^, EnSVM uses several linear SVMs to classify fragments represented by different *k*-mers, one *k*-mer size for one SVM. Then, EnSVM uses majority vote, a simply and widely used aggregation technique, to integrate the predictions of these SVMs and to divide fragments into the confident set and diffident set. Fragments in the confident set are considered as consistent predictions since they get more than voting threshold same predictions with respect to their species by these linear SVMs, whereas fragments in the diffident set are considered as inconsistent predictions, since they get fewer than voting threshold same predictions. In fact, researchers can use a user-specified threshold value to produce these two sets. If the value is set as 3 and a fragment obtains at least 3 consistent predictions from these SVMs, this fragment is put into the confident set; otherwise, it is placed into the diffident set by EnSVM. Figure 4a shows the flowchart of EnSVM.

### EnSVMB

Fragments in the confident set can be accurately classified by EnSVM, but not so for fragments in the diffident set. To improve the true positives and specificity on the diffident set, EnSVMB further resorts to BLAST to reclassify fragments in the diffident set. Each fragment in the diffident set is used as a query sequence for BLAST and the reference database of BLAST is the same as the training set of EnSVM. However, for a fragment in the diffident set, BLAST sometimes cannot find out a reference sequence with the best hit and confident *e*-value. EnSVMB tags this fragment as unknown. In the end, EnSVMB combines predictions on the diffident set and those on the confident set, and reports overall performance on all fragments in the validation set, except the ones tagged with unknown. Figure 4b shows the flowchart of EnSVMB.

We want to remark that, although these five adopted SVMs are linear classifiers, EnSVM and EnSVMB are nonlinear classifiers^26, 27^, so they share the advantage of non-linear SVM^28^. These five linear SVMs are independent and trained in parallel. The number of fragments in the diffident set is much smaller than that in the confident set, BLAST on the diffident set is much faster than on the confident set. Given that, EnSVMB can hold the advantage of ensemble classifier and keep the similar runtime cost as a linear SVM.

### Linear SVM

We adopt linear SVM as the base classifier for EnSVM and EnSVMB. We use multicore LIBLINEAR^21, 29, 30^ package for parallel training SVM and one-vs-rest strategy^31^ to solve multi-class problem.

Suppose ${x}_{j}\in {{\mathbb{R}}}^{D}$($D={4}^{k}$) is the input data, $j\in \mathrm{\{1,}\cdots ,N\}$ and *N* is the number of fragments in the reference (or training) set. The objective functions of linear SVM is:

1 $$\begin{array}{c}\mathop{min}\limits_{W,{\xi }_{j}}\frac{1}{2}{W}^{T}W+\omega \sum _{j=1}^{N}{\xi }_{j}\\ s\mathrm{.}t\mathrm{.}\,{W}^{T}{x}_{j}{c}_{j}\ge 1-{\xi }_{j}\,\forall j\\ \quad \quad \quad \,{\xi }_{j}\ge 0\,\forall j\end{array}$$

where ${c}_{j}\in \{-\mathrm{1,}+1\}$ is the species label for the *j*-th fragment. If the *j*-th fragment belongs to the *c*-th species, then ${c}_{j}=1$; otherwise, ${c}_{j}=-1$. $\omega$ is a scalar parameter to control the loss of constraints violation and ${\xi }_{j}$ is a slack variable to penalize the *j*-th fragment if it violates the margin requirement.

LIBLINEAR supports L2-loss and L1-loss linear SVM. In this paper, we use an L2-loss SVM as follows:

2 $$\mathop{min}\limits_{W}\frac{1}{2}{W}^{T}W+\omega \sum _{j\mathrm{=1}}^{N}max{\mathrm{(1}-{W}^{T}{x}_{j}{c}_{j},\mathrm{0)}}^{2}$$

### BLAST

Although BLAST is an efficient local alignment based tool, it still asks for long runtime to process a large amount of fragments. Since a small portion of fragments are divided into the diffident set by EnSVM, EnSVMB only uses BLAST (blastn^20^) to deal with these fragments. To reduce the runtime of BLAST, EnSVMB uses shell script to run BLAST in parallel and thus the runtime cost of integrating BLAST with EnSVM can be further reduced. The reference database for BLAST is the same with the reference set, which is used to train SVMs.

## Conclusion

We proposed a new approach called EnSVM and its advanced method EnSVMB for classifying metagenomics fragments. EnSVM firstly trains five linear SVMs with respect to different *k*-mers to explore and exploit the complementary information between these *k*-mers. Then it divides fragments into a confident set and a diffident set based on the aggregated predictions from these SVMs. The accuracy, specificity and sensitivity of EnSVM on confident set are much higher than those on diffident set. To improve the accuracy, specificity and sensitivity on diffident set, EnSVMB applies BLAST to reclassify fragments in that set. Experiments show that EnSVM can effectively and accurately pick out hard-to-be classified fragments and EnSVMB can more accurately classify fragments than other related methods. EnSVMB does not significantly increase the runtime cost, but significantly improves the overall performance (accuracy, sensitivity, specificity and number of true positives). EnSVMB is an accurate and yet efficient approach for metagenomics fragments classification.

## Electronic supplementary material

Supplementary file
