# Supplementary material for: EnSVMB: Metagenomics Fragments Classification using Ensemble SVM and BLAST
Source: Sci Rep. 2017 Aug 25;7:9440. doi: 10.1038/s41598-017-09947-y (PMC5573435; doi:10.1038/s41598-017-09947-y)
Supplement: Supplementary file 1 — Supplementary file [file 41598_2017_9947_MOESM1_ESM.pdf]

Supplementary file of 'EnSVMB: Metagenomics Fragments Classification using Ensemble SVM and BLAST'

Yuan Jiang [1], Jun Wang [1], Dawen Xia [2,3], Guoxian Yu [1,*]
[1] College of Computer and Information Science, Southwest University, Chongqing, China;
[2] College of Data Science and Information Engineering, Guizhou Minzu University, Guiyang, China;
[3] College of National Culture and Cognitive Science, Guizhou Minzu University, Guiyang, China;

July 5, 2017

Species in the medium dataset, large dataset and real gut metagenome
69 microbial species used in the medium dataset.

Table S1: Species in the medium dataset.

Streptococcus salivarius	Leptospira interrogans serovar	Mycobacterium abscessus
Streptococcus equi	Leptospira borgpetersenii serovar	Methylobacterium extorquens
Burkholderia mallei	Mycobacterium leprae	Lactobacillus rhamnosus
Streptococcus intermedius	Rhodococcus erythropolis	Neisseria gonorrhoeae
Enterococcus faecalis	Streptomyces hygroscopicus	Bordetella bronchiseptica
Helicobacter cetorum	Campylobacter coli	Bordetella pertussis
Bacillus coagulans	Campylobacter fetus	Enterobacter aerogenes
Paenibacillus pkoymyxa	Mycoplasma gallisepticum	Erwinia amylovora
Clostridium acetobutylicum	Mycoplasma mycoides	Klebsiella oxytoca
Clostridium perfringens	Mycoplasma leachii	Shigella boydii
Clostridium tetani	Ruminococcus obeum	Yersinia enterocolitica
Clostridium thermocellum	Helicobacter cinaedi	Vibrio parahaemolyticus
Clostridium kluyveri	Methanosarcina mazei	Rickettsia canadensis
Spirochaeta thermophila	Methanosarcina acetivorans	Desulfovibrio vulgaris
Caulobacter crescentus	Shewanella putrefaciens	Desulfovibrio vulgaris
Lactobacillus acidophilus	Thermus thermophilus	Ehrlichia canis str
Lactobacillus buchneri	Arcobacter butzleri	Chlamydia trachomatis
Lactobacillus delbrueckii	Staphylococcus pseudintermedius	Bacteroides fragilis
Lactobacillus helveticus	Rhizobium etli	Bacteroides vulgatus
Lactobacillus fermentum	Brucella melitensis	Serratia plymuthica
Corynebacterium diphtheriae	Brucella suis	Prevotella ruminicola
Bifidobacterium bifidum	Brucella abortus	Desulfovibrio africanus
Bifidobacterium breve	Xanthomonas campestris	Xanthomonas oryzae

331 microbial species used in the large dataset.

*Corresponding author: email: gxyu@swu.edu.cn, Tel.: +86-23-6825-4396.

Table S2: Species in the large dataset.

Cupriavidus necator	Rhodospirillum rubrum	Chlorobium phaeobacteroides
Brucella ceti	Leuconostoc mesenteroides	Staphylococcus aureus
Staphylococcus epidermidis	Streptococcus salivarius	Streptococcus suis
Streptococcus thermophilus	Streptococcus mutans	Streptococcus agalactiae
Streptococcus pneumoniae	Streptococcus pyogenes	Streptococcus parasanguinis
Streptococcus anginosus	Streptococcus dysgalactiae	Streptococcus equi
Burkholderia mallei	Streptococcus intermedius	Enterococcus faecalis
Cupriavidus metallidurans	Thermotoga thermarum	Marinomonas mediterranea
Geobacter daltonii	Mycobacterium yongonense	Brucella pinnipedialis
Pseudomonas sp. UW4	Pyrobaculum arsenaticum	Streptomyces rapamycinicus
Enterococcus faecium	Helicobacter cetorum	Borrelia burgdorferi
Bacillus amyloliquefaciens	Bacillus anthracis	Bacillus cereus
Bacillus coagulans	Bacillus licheniformis	Bacillus megaterium
Paenibacillus polymyxa	Bacillus subtilis	Bacillus thuringiensis
Clostridium acetobutylicum	Clostridium botulinum	Clostridium perfringens
Clostridium tetani	Ruminiclostridium thermocellum	Burkholderia ambifaria
Clostridium kluyveri	Spirochaeta thermophila	Caulobacter crescentus
Lactobacillus acidophilus	Lactobacillus brevis	Lactobacillus buchneri
Lactobacillus casei	Lactobacillus delbrueckii	Lactobacillus helveticus
Serratia sp. ATCC 39006	Frankia sp. CcI3	Thermoanaerobacter italicus
Burkholderia sp. YI23	[Cellvibrio] gilvus	Sinorhizobium medicae
Pyrobaculum sp. 1860	Rickettsia philipii	Mycobacterium vanbaalenii
Lactobacillus plantarum	Lactobacillus reuteri	Treponema pallidum
Lactobacillus amylovorus	Lactobacillus fermentum	Anaeromyxobacter dehalogenans
Lactobacillus salivarius	Listeria monocytogenes	Bifidobacterium bifidum
Bifidobacterium breve	Corynebacterium diphtheriae	Corynebacterium glutamicum
Corynebacterium pseudotuberculosis	Leptospira biflexa	Leptospira interrogans
Leptospira borgpetersenii	Propionibacterium acnes	Mycobacterium avium
Mycobacterium bovis	Mycobacterium intracellulare	Mycobacterium leprae
Mycobacterium smegmatis	Mycobacterium tuberculosis	Mycobacterium gilvum
Rhodococcus erythropolis	Desulfovibrio hydrothermalis	Streptomyces hygroscopicus
Campylobacter coli	Campylobacter fetus	Campylobacter jejuni
Mycoplasma gallisepticum	Helicobacter pylori	Mycoplasma mycoides
Mycoplasma leachii	Mycoplasma fermentans	Helicobacter cinaedi
Bifidobacterium longum	Methanosarcina mazei	Pyrococcus furiosus
Rhodococcus pyridinivorans	Clostridium sp. SY8519	Thermococcus sp. 4557
Sulfolobus acidocaldarius	Sulfolobus solfataricus	Brucella abortus
Shewanella putrefaciens	Francisella tularensis	Burkholderia cenocepacia
Thermus thermophilus	Marinobacter aquaeolei	Bifidobacterium animalis
Staphylococcus lugdunensis	Arcobacter butzleri	Staphylococcus pseudintermedius
Rhodospirillum photometricum	Chlorobium limicola	Chlorobium phaeovibrioides
Burkholderia pseudomallei	Pseudomonas aeruginosa	Pseudomonas fluorescens
Rhizobium etli	Brucella melitensis	Brucella suis
Mycoplasma haemofelis	Pseudomonas mendocina	Pseudomonas putida
Streptococcus gallolyticus	Pseudomonas stutzeri	Synechococcus elongatus
Xanthomonas campestris	Lactobacillus johnsonii	Rickettsia bellii
Candidatus Liberibacter	Xanthomonas axonopodis	Xanthomonas oryzae
Geobacter sulfurreducens	Rickettsia massiliae	Rickettsia slovaca
Mycobacterium abscessus	Brucella canis	Sinorhizobium fredii
Pseudomonas protegens	Sinorhizobium meliloti	Rhizobium leguminosarum
Methanococcus maripaludis	Eubacterium rectale	[Eubacterium] siraeum
Methylobacterium extorquens	Sulfolobus islandicus	Corynebacterium urealyticum
Acinetobacter baumannii	Lactobacillus rhamnosus	Neisseria gonorrhoeae
Neisseria meningitidis	Desulfitobacterium hafniense	Bordetella bronchiseptica
Bordetella parapertussis	Bordetella pertussis	Brachyspira pilosicoli
Hyphomicrobium denitrificans	Enterobacter aerogenes	Enterobacter cloacae
Geobacillus sp. JF8	Streptococcus iniae SF1	Actinoplanes sp. SE50 110
Erwinia amylovora	Pantoea ananatis	Pectobacterium carotovorum
Vibrio anguillarum	Klebsiella oxytoca	Klebsiella pneumoniae
Burkholderia thailandensis	Serratia marcescens	Paenibacillus mucilaginosus
Shigella boydii	Shigella dysenteriae	Shigella flexneri
Shewanella baltica	Shigella sonnei	Yersinia enterocolitica
Yersinia pestis	Yersinia pseudotuberculosis	Edwardsiella tarda
Aeromonas hydrophila	Corynebacterium ulcerans	Francisella noatunensis
Vibrio cholerae	Vibrio parahaemolyticus	Vibrio vulnificus
Actinobacillus pleuropneumoniae	Haemophilus influenzae	Haemophilus parasuis
Pseudomonas monteilii	Streptococcus constellatus	Anaplasma marginale
Wolbachia endosymbiont	Ehrlichia ruminantium	Rickettsia prowazekii
Rickettsia rickettsii	Mycobacterium canettii	Rickettsia typhi
Rickettsia canadensis	Erwinia pyrifoliae	Bartonella quintana
Chlamydia trachomatis	Bacteroides fragilis	Serratia plymuthica
Chlamydia psittaci	Chlamydophila pneumoniae	Chlamydia pecorum
Desulfovibrio desulfuricans	Desulfovibrio vulgaris	Acidithiobacillus ferrooxidans
Pyrococcus yayanosii	Desulfotomaculum gibsoniae	Streptococcus infantarius
Marinobacter adhaerens	Thioalkalivibrio sulfidiphilus	Nostoc sp. PCC
Synechococcus sp. CC9605	Chloroflexus aurantiacus	Thermus sp. CCB
Vibrio sp. EJY3	Arthrobacter sp. Rue61a	Sulfolobus tokodaii str. 7
Bacillus sp. JS	Sphaerochaeta globosa str. Buddy	Sphaerochaeta pleomorpha str. Grapes
Bradyrhizobium sp. ORS 278	Methylobacterium nodulans	Acinetobacter oleivorans DR1
Streptococcus sp. I-G2	Streptococcus sp. I-P16	Leuconostoc gasicomitatum
Ruminococcus champanellensis	Anabaena cylindrica	Pectobacterium sp. SCC3193
Enterobacter sp. R4-368	Mycobacterium sp. MOTT36Y	Streptomyces pratensis
Anabaena variabilis	Synechococcus sp. PCC 7502	Methanocella conradii
Methanocella arvoryzae	Agrobacterium fabrum	Pyrococcus sp. ST04
Nitrosococcus oceani	Candidatus Nitrosopumilus sp. AR2	Geobacillus sp. GHH01
Leuconostoc gelidum	Leuconostoc carnosum	Edwardsiella piscicida
Ruminococcus albus	Streptomyces sp. PAMC26508	Carnobacterium sp. WN1359
Thermotoga sp. RQ2	Liberibacter crescens	Staphylococcus carnosus
Staphylococcus haemolyticus	Staphylococcus warneri	Deinococcus radiodurans
Streptococcus gordonii	Streptococcus oralis	Streptococcus sanguinis
Spirochaeta sp. L21-RPul-D2	Archaeoglobus sulfaticallidus	Rhodococcus jostii
Exiguobacterium antarcticum	Enterobacter lignolyticus	Nitrosococcus halophilus
Streptococcus parauberis	Streptococcus uberis	Enterococcus hirae
Nocardia cyriacigeorgica	Bradyrhizobium diazoefficiens	Leuconostoc kimchii
Pyrobaculum aerophilum	Methanobacterium sp. MB1	Serratia symbiotica
Exiguobacterium sp. MH3	Borrelia hermsii	Bacillus pumilus
Bacillus cellulosilyticus	Pseudomonas sp. TKP	Geobacillus thermoglucosidasius
Hyphomicrobium nitrativorans	Bacillus atrophaeus	Geobacillus kaustophilus
Paenibacillus larvae	Burkholderia phymatum	Clostridium cellulovorans
Streptococcus lutetiensis	Clostridium pasteurianum	Vibrio sp. Ex25
Treponema azotonutricium	[Clostridium] stercorarium	Candidatus Nitrosopumilus koreensis AR1
Clostridium beijerinckii	Clostridium cellulolyticum	Chloroflexus aggregans
Corynebacterium efficiens	Clostridium ljungdahlii	Nitrosomonas sp. AL212
Clostridium novyi	Bacillus toyonensis	Desulfosporosinus orientis
Desulfotomaculum ruminis	Treponema denticola	Brachyspira hyodysenteriae
Lactobacillus gasseri	Lactobacillus paracasei	Paenibacillus terrae
Lactobacillus sakei	Acidithiobacillus ferrivorans	Corynebacterium kroppenstedtii
Lactobacillus ruminis	Lactobacillus sanfranciscensis	Thermococcus sp. CL1
Wolbachia endosymbiont of Drosophila melanogaster	Listeria ivanovii	Listeria seeligeri
Listeria innocua	Listeria welshimeri	Cupriavidus taiwanensis
Anaplasma phagocytophilum		

80 microbial species used as the reference set for experiments on the real gut metagenome.

Table S3: Species in the real gut genome dataset.

Prevotella copri	Faecalibacterium prausnitzii	Prevotella stercorea
Blautia producta	Ruminococcus gnavus	Dorea formicigenerans
Rhodococcus pyridinivorans	Thioalkalivibrio sulfidiphilus	Chlorobium limicola
Streptococcus salivarius	Leptospira interrogans serovar	Mycobacterium abscessus
Streptococcus equi	Leptospira borgpetersenii serovar	Methylobacterium extorquens
Burkholderia mallei	Mycobacterium leprae	Lactobacillus rhamnosus
Streptococcus intermedius	Rhodococcus erythropolis	Neisseria gonorrhoeae
Enterococcus faecalis	Streptomyces hygroscopicus	Bordetella bronchiseptica
Helicobacter cetorum	Campylobacter coli	Bordetella pertussis
Bacillus coagulans	Campylobacter fetus	Enterobacter aerogenes
Paenibacillus pkoymyxa	Mycoplasma gallisepticum	Erwinia amylovora
Clostridium acetobutylicum	Mycoplasma mycoides	Klebsiella oxytoca
Clostridium perfringens	Mycoplasma leachii	Shigella boydii
Clostridium tetani	Ruminococcus obeum	Yersinia enterocolitica
Clostridium thermocellum	Helicobacter cinaedi	Vibrio parahaemolyticus
Clostridium kluyveri	Methanosarcina mazei	Rickettsia canadensis
Spirochaeta thermophila	Methanosarcina acetivorans	Desulfovibrio vulgaris
Caulobacter crescentus	Shewanella putrefaciens	Desulfovibrio vulgaris
Lactobacillus acidophilus	Thermus thermophilus	Ehrlichia canis str
Lactobacillus buchneri	Arcobacter butzleri	Chlamydia trachomatis
Lactobacillus delbrueckii	Staphylococcus pseudintermedius	Bacteroides fragilis
Lactobacillus helveticus	Rhizobium etli	Bacteroides vulgatus
Lactobacillus fermentum	Brucella melitensis	Serratia plymuthica
Corynebacterium diphtheriae	Brucella suis	Prevotella ruminicola
Bifidobacterium bifidum	Brucella abortus	Desulfovibrio africanus
Bifidobacterium breve	Xanthomonas campestris	Xanthomonas oryzae
Rhodospirillum photometricum	Rickettsia philipii	
